# Genome Sequence of a Distinct Infectious Bursal Disease Virus

**DOI:** 10.1128/genomeA.01061-15

**Published:** 2015-10-01

**Authors:** Gonzalo Tomás, Martín Hernández, Ana Marandino, Diego Hernández, Claudia Techera, Sofía Grecco, Yanina Panzera, Ruben Pérez

**Affiliations:** Facultad de Ciencias, Sección Genética Evolutiva, Instituto de Biología, Universidad de la República, Montevideo, Uruguay

## Abstract

Infectious bursal disease virus is a relevant avian pathogen that affects poultry production. Here, we report the full-length coding sequence of the Uruguayan strain dIBDV/UY/2014/2202, isolated from a commercial broiler flock. The strain belongs to the distinct IBDV lineage that is widely distributed in South America.

## GENOME ANNOUNCEMENT

Infectious bursal disease virus (IBDV) belongs to the genus *Avibirnavirus* within the family *Birnaviridae*. This nonenveloped icosahedral virus has a double-stranded RNA genome consisting of two segments named A and B (1).

IBDV is a highly contagious avian pathogen affecting commercial poultry production worldwide (2). All pathogenic IBDVs (serotype 1) can be divided into classic, variant, and very virulent strains according to antigenic and pathogenic criteria (3). Phylogenetic analyses have consistently recovered the clades or lineages corresponding to these strains, including a clade composed of vaccine-like strains and a recently described distinct global lineage (4). This distinct lineage (dIBDV) is widely distributed in South America (4, 5).

The IBDV strain was collected in 2014 from a 27-day-old commercial broiler flock suffering from respiratory problems and increased mortality. Viral RNA was isolated from bursae tissue using TRIzol reagent (Invitrogen). First-strand cDNA was synthesized using random hexamers (Thermo Scientific). The IBDV was diagnosed as a non-very virulent virus by real-time PCR (6). The complete genome sequence of this isolate was obtained by reverse transcription PCR, using overlapped consensus primers and direct sequencing. Purified products were sequenced in both directions by Macrogen Inc. (Seoul, Republic of Korea). Sequences were compiled and edited using the SeqMan program (Lasergene). Multisequence alignments were performed with MEGA5 (7), and phylogenetic trees were constructed using PhyML.

The coding region of segment A (3,073 nucleoides) contains two open reading frames that encode VP5 (145 aa) and a VP2-VP4-VP3 polyprotein (1,012 aa); segment B encodes VP1 (879 aa), the viral RNA polymerase.

The phylogenetic analysis of the VP2 hypervariable region indicates that the virus belongs to the dIBDV lineage. This was confirmed by the presence of a unique and conserved molecular signature (272T, 289P, 290I, and 296F) that is a diagnostic character for the classification of dIBDVs (4). Segment B also associates with strains of the dIBDV lineage and shows the 243P diagnostic marker. Consequently, the strain was denoted as dIBDV/UY/2014/2202. This is the first full-length sequence of the coding genome of a strain belonging to the dIBDV lineage.

Comparative analysis of the complete segment A shows higher nucleotide similarity (96%) with classical strains (e.g., Edgar and HPR-2). In the case of VP1 (segment B), the highest similarity (96.4%) was found with strains that are not very virulent (e.g., JD1 and Irwin Moulthrop).

The comparison of the VP2 hypervariable region of dIBDV/UY/2014/2202 with most vaccine strains commonly used in South America showed an amino acid similarity ranging from 88.9 to 92.1%; D78 (Nobilis D78) and Winterfield 2512 (Cevac IBD L) were the most similar strains.

A comprehensive study of more genomic sequences of these dIBDVs is needed to understand the virus’s epidemiology and to contribute to the effective control of IBDV infection.

### Nucleotide sequence accession numbers.

The full-length coding sequence of strain dIBDV/UY/2014/2202 has been deposited in GenBank under the accession numbers KT336459 (segment A) and KT336458 (segment B).
